# Opinion: New perspectives on willingness to communicate in a second language

**DOI:** 10.3389/fpsyg.2022.998839

**Published:** 2022-09-13

**Authors:** Dianlei Geng, Xiuling Wang

**Affiliations:** School of Foreign Languages, Wuhan University of Science and Technology, Wuhan, China

**Keywords:** new perspectives, willingness to communicate, second language, theoretical approaches, methodologies

Since the concept of willingness to communicate (WTC) was introduced into research on second language (L2) teaching by Maclntyre and Charos (1996), it has become a hot spot in the field of applied linguistics, particularly its sub-field of SLA. To date, researchers in L2 WTC have used diverse methodologies and theoretical perspectives to reveal factors that promote or hinder learners' WTC. However, some issues warrant further investigations, which can be illustrated by a few examples, as follows: What is the subtle dynamic change process of communicative intention that is complex, ever-changing, and affected by a number of intermeshing factors? Are there any differences in WTC between learners of English and other languages? How can we better understand the dynamic nature of communicative intention in depth? Answers to these questions, and in fact many more, are well provided in the book under review, i.e., *New Perspectives on Willingness to Communicate in a Second Language* (Zarrinabadi and Pawlak, 2021).

As a volume of the book series *Second Language Learning and Teaching*, this book encompasses a wide range of topic-specific chapters on learners' WTC in L2 and presents methodological approaches including qualitative, quantitative, and mixed methods. Overall, the book serves to study in depth how culture and technology influence WTC in L2.

The main thrust of this volume contains 11 main chapters (Chapters 2-12), which probe into L2 WTC from different perspectives.

Of all, Chapters 2, 3, and 10 are devoted to theoretical perspectives of L2 WTC. Chapter 2 discusses three definitions of WTC and conceptualizes the key properties of complex dynamic systems (CDS). It also identifies the degree of compatibility between WTC and the CDS, proving that the former can be viewed as a complex dynamic system. Firstly, WTC is strongly dependent on initial conditions (i.e., learners' age, their proficiency in a second language, etc.). Secondly, the changes in different variables that affect WTC are “self-organized,” “non-linear” and “idiosyncratic.” Then, the subsystems of WTC are interrelated, and when the subsystems (i.e., cognitive factors, emotional factors, etc.) that affect WTC are stable at a certain point of time, the individual's WTC will be in an attractor state. However, once these subsystems experience any change or reorganization, the stability of WTC will be interrupted. Therefore, the theory of CDS invites researchers to look into the potential changing processes of WTC, and the complex interaction among a plethora of factors in the WTC system, which is helpful for teachers to address situated questions and issues in their local classroom context. Based on the ecosystems framework, Chapter 3 makes a comprehensive analysis of the contributing factors from micro-classroom level to macro-social level, and further illustrates the complexity of these factors. This ecological stance, which features a holistic view and attempts to bring into consideration the whole language learning ecology (a complex set of interwoven factors) (Steffensen and Kramsch, 2017), is helpful for teachers to create a supportive environment inside and outside the classroom for developing learners' WTC. Different from Chapters 2 and 3, Chapter 10 mainly analyzes the concept of “opportunity” within WTC by social network approach, which links the learners' WTC with the social relations around them. What all these theoretical perspectives share is the emphasis on the multiplicity of factors that combine to influence students' WTC, and the complex interaction of these factors that contributes to the dynamism of WTC.

Chapter 4 and Chapter 5, cutting in from a cultural perspective, both focus on the relationship between cultural factors and L2 WTC. But the two chapters demonstrate their distinctive characteristics. Chapter 4 attempts to unearth German expats' WTC in German rather than in English, where it is concluded that the expats' situation in cultural immersion in Germany greatly enhanced their WTC in German. In contrast, English as a lingua franca also negatively influenced expats' WTC in German, because some native English speakers in German believed that English was sufficient for communication, and therefore learning German was not necessary. Besides, other factors are reported to have influenced the expats' WTC in German, including their nationality, education, language skills, age, gender, and occupation. From a different angle, Chapter 5 examines the mediating role of culture between students' foreign language self-assessment and L2 WTC based on the Italian and Polish EFL contexts. It attests that the strength of the relationship between L2 WTC and foreign language self-assessment depends on the students' cultural background.

Chapter 6 and Chapter 11 both concern the factors that influence L2 learners' WTC from the perspective of teachers. Based on attribution theory, Chapter 6 is committed to exploring teachers' views on students' WTC and reticence. This study purposefully selected six English teachers from a private language school in Iran, and used semi-structured interviews for investigation. As the study reveals, the teachers interviewed were generally passive toward the reticent students, and they all perceived reticence as the result of students' internal causes— a stereotype formed over the teachers' years of teaching and learning. Chapter 11, using the questionnaire data of 220 EFL students and 24 English teachers in a private language institute in Iran, illustrates the effects of teacher's “self-disclosure,” “immediacy,” and “technology policy” (teacher's regulations on students' use of wireless communication technology in class) on WTC of Iranian intermediate English students. The findings manifest that these teacher-related factors are all positive predictors of students' WTC.

Chapter 7 explores the impact of extroversion on L2 WTC through a questionnaire survey on 494 students from 20 classes randomly selected by six grammar schools in a town of Poland. It is discovered that extroversion had no direct effect on L2 WTC, but it could indirectly affect learners' WTC by influencing their perception of language competence and language anxiety. It is argued, however, that a high level of extroversion is probably beneficial to foreign language learning.

In Chapter 8, mixed methods are used to investigate the influence of the flipped classroom on learners' WTC. In an experimental study, a flipped classroom and a conventional English classroom were set up as the experimental group and the control group, respectively. In combination with the experimental data, semi-structured interviews were also employed to further explain how the flipped classroom enhances learners' L2 WTC. In the conventional English classroom, students do not know what they are going to learn until they are in class and are ignorant of the classroom assignment, while in a flipped classroom, students are familiar with the learning materials and the general flow of the teaching processes before the class. In addition, the flipped classroom exposes students to various interesting learning resources (videos, pictures, etc.), which makes students more motivated to learn. The research results show that sufficient preparation before class reduced students' in-class anxiety. Besides, teachers' hard preparation for pre-class materials also made students feel valued so that they respected teachers' good intentions and had higher learning motivation. What's more, this research confirms the effectiveness of the flipped classroom and makes up for the gap of research on the influence of computer-assisted environments on WTC.

Chapter 9 takes 38 freshmen in an Iranian university as research objects, trying to reveal the dynamic relationship among WTC, anxiety and enjoyment by employing the experience sampling method. The research exhibits that the relationship among the three variables changed significantly during the weekly class meeting or from 1 week to another. Furthermore, by analyzing the moving correlations among the above three variables, the research also elucidates in detail that there existed a strong and positive correlation between enjoyment and WTC vis-à-vis a negative one between WTC and anxiety as well as between enjoyment and anxiety.

Chapter 12 centers on the effect of L2 vocabulary size on L2 WTC of 100 EFL students at a state university in Turkey. The research used instruments such as WTC inside the Classroom Scale and Vocabulary Levels Test, and disclosed a vital correlation between participants' vocabulary level and their WTC in class. The findings indicate that accumulating rich vocabulary will promote students' confidence in communication, thus enhancing their WTC in class. The study also suggests that longitudinal research can be conducted to observe learners' development of vocabulary level and L2 WTC.

Toward the end of the volume, the concluding chapter summarizes the directions for future research in L2 WTC. First, it is essential to probe into whether people are willing to communicate with other skills than “speaking,” such as writing, listening, and reading. Second, different classroom practices and teaching skills should be examined so as to improve L2 WTC. Third, the relationship between psychological intervention and WTC cannot be ignored. Fourth, learners' WTC in a third language or the X language deserves further exploration. Fifth, the relationship between computer technology and WTC is also worthy of further attention.

In this book, studies on L2 WTC are mainly focused on case scenarios in western countries, but they are rightfully coupled with a few studies on Asian countries, which provide good inspiration for readers to see the big picture. Of all, Asian students' silence in class has been of great concern. It is believed that this phenomenon results from Asian students' personality (shyness, nervousness, etc.) (Hsu, 2015), classroom atmosphere (Wang and Cheng, 2017), and traditional Confucian culture (Jackson, 2003), etc. Some chapters in this volume shed some light on these issues. To illustrate, Chapter 6 shows that some teachers treat reticent students with a negative attitude, but teachers can, in effect, contribute to students' classroom participation by creating a positive classroom environment (Long and Xu, 2020). In the first place, teachers should not stereotype Asian students as reticent and passive learners, but treat them equally (Shi, 2021), and then students in turn are more likely to take a more active part in the class. In a similar vein, Chapter 11 highlights that teachers' friendly behavior and self-disclosure can narrow the distance between teachers and students and then enhance students' WTC by making them not feel nervous or shy. All in all, teachers should show more concern for reticent students and establish a good teacher-student relationship (Long et al., 2021).

Overall, *New Perspectives on Willingness to Communicate in a Second Language* provides new theoretical perspectives for the study of L2 WTC, such as the complex dynamic system theory and the social network approach. Larsen-Freeman (2008) contends that CDS theory is concerned with the dynamic changing tendencies of the whole system instead of the static single causality. It directs researchers of WTC to focus on the dynamic changes and the relationships between variables. The social network approach, on the other hand, enables the study of WTC to center on the multi-level analysis of the interaction between individuals and their surrounding social relationships, thereby expanding the relevant research from the micro-personal level to the more systematic social relationship level. In addition, a wealth of data collection and analytical methods, as well as different forms of questionnaires in this book will provide powerful tools for readers' future research. These methods are beneficial for researchers to uncover the complex, dynamic, and multifaceted nature of L2 WTC from multiple perspectives.

Despite all the above strengths that the book well deserves, readers may find it more comprehensive if it included a chapter dwelling upon the L2 WTC of students in primary or preschool education. This might be a powerful supplement to the study of L2 WTC in the future.

Notwithstanding, the profound strengths overshadow the weakness in this book. This volume is a valuable addition to the literature in the field of SLA or applied linguistics at large. It is worth recommending to students, teachers, and researchers in the relevant domain.

## Author contributions

XW drafted the initial manuscript. DG helped XW to select the book, provided insights and suggestions during her writing, and finally made substantial revision on the last draft. Both authors contributed to the article and approved the submitted version.

## Funding

This article was supported by: the Hubei Provincial Key Research Project for Philosophy and Social Sciences in Higher Learning Institutions (Grant Number: 21D008); and, the Incubation Project of High-level Humanities and Social Sciences of Wuhan University of Science and Technology (Grant Number: W201907).

## Conflict of interest

The authors declare that the research was conducted in the absence of any commercial or financial relationships that could be construed as a potential conflict of interest.

## Publisher's note

All claims expressed in this article are solely those of the authors and do not necessarily represent those of their affiliated organizations, or those of the publisher, the editors and the reviewers. Any product that may be evaluated in this article, or claim that may be made by its manufacturer, is not guaranteed or endorsed by the publisher.
